# Association between Polymorphisms of Hemochromatosis (HFE), Blood Lead (Pb) Levels, and DNA Oxidative Damage in Battery Workers

**DOI:** 10.3390/ijerph20043513

**Published:** 2023-02-16

**Authors:** Willian Robert Gomes, Paula Pícoli Devóz, Bruno Alves Rocha, Denise Grotto, Juliana Mara Serpeloni, Bruno Lemos Batista, Alexandros G. Asimakopoulos, Kurunthachalam Kannan, Fernando Barbosa Jr., Gustavo Rafael Mazzaron Barcelos

**Affiliations:** 1 Department of Clinical Analyses, Toxicology and Food Sciences, School of Pharmaceutical Sciences of Ribeirão Preto, University of São Paulo, Avenida do Café s/n, Ribeirão Preto 14040-903, Brazil; 2 University of Sorocaba, Rodovia Raposo Tavares, km 92.5, Sorocaba 18023-000, Brazil; 3 Department of General Biology, State University of Londrina, Rodovia Celso Garcia Cid, km 380, Londrina 86057-970, Brazil; 4 Center of Natural and Human Sciences, Federal University of ABC, Avenida dos Estados, 5001, Santo André 09210-580, Brazil; 5 Department of Chemistry, Faculty of Natural Sciences, Norwegian University of Science and Technology, Realfagbygget, D2-163, Gløshaugen, Høgskoleringen 5, 7491 Trondheim, Norway; 6 Department of Pediatrics and Environmental Medicine, New York University School of Medicine, New York, NY 10016, USA; 7 Bioactive Natural Products Research Group, Department of Biochemistry, Faculty of Science, King Abdulaziz University, Jeddah 21589, Saudi Arabia; 8 Department of Biosciences, Institute for Health and Society, Federal University of São Paulo, Rua XV de Novembro, 195, Santos 11101-151, Brazil

**Keywords:** biomonitoring, DNA damage, HFE C282Y, HFE H63D, 8-hydroxy-2′-deoxyguanosine, toxicity

## Abstract

Occupational exposure to lead (Pb) continues to be a serious public health concern and may pose an elevated risk of genetic oxidative damage. In Brazil, car battery manufacturing and recycling factories represent a great source of Pb contamination, and there are no guidelines on how to properly protect workers from exposure or to dispose the process wastes. Previous studies have shown that Pb body burden is associated with genetic polymorphisms, which consequently may influence the toxicity of the metal. The aim of this study was to assess the impact of Pb exposure on DNA oxidative damage, as well as the modulation of hemochromatosis (HFE) polymorphisms on Pb body burden, and the toxicity of Pb, through the analysis of 8-hydroxy-2′-deoxyguanosine (8-OHdG), in subjects occupationally exposed to the metal. Male Pb-exposed workers (n = 236) from car battery manufacturing and recycling factories in Brazil participated in the study. Blood and plasma lead levels (BLL and PLL, respectively) were determined by ICP-MS and urinary 8-OHdG levels were measured by LC-MS/MS, and genotyping of HFE SNPs (rs1799945, C → G; and 1800562, G → A) was performed by TaqMan assays. Our data showed that carriers of at least one variant allele for HFE rs1799945 (CG + GG) tended to have higher PLL than those with the non-variant genotype (β = 0.34; *p* = 0.043); further, PLL was significantly correlated with the levels of urinary 8-OHdG (β = 0.19; *p* = 0.0060), while workers that carry the variant genotype for HFE rs1800562 (A-allele) showed a prominent increase in 8-OHdG, as a function of PLL (β = 0.78; *p* = 0.046). Taken together, our data suggest that HFE polymorphisms may modulate the Pb body burden and, consequently, the oxidative DNA damage induced by the metal.

## 1. Introduction

Human exposure to lead (Pb) and its compounds occurs mainly in occupational settings, especially in industrial processes such as smelting, pottery, shipbuilding, Pb-based painting, Pb-containing pipes, car battery recycling, grids, firearms industry, pigments, printing, among others [1]. Some other sources, such as canned food, cosmetics, lead pipes used in water supply, and the use of certain herbal products, can also contribute to Pb exposure in humans [2]. McFarland et al. [3] showed that humans may also have underpredicted high-lead exposures owing to leaded paints and pipes, which tend to aggregate within communities with high rates of homes with lead service lines and lead paint in disrepair.

In Brazil, car battery manufacturing and recycling activities represent a major source of Pb contamination, and the country still lacks guidelines on the management of end-of-life Pb-acid batteries and has no law defining the collection and recycling of this type of waste [4]. The Brazilian National Institute of Metrology Standardization and Industrial Quality (INMETRO) included automotive batteries in a compulsory certification program as this represents a high risk to the environment and human health [5].

It is well-established that, following exposure, Pb interferes with enzymatic and non-enzymatic components of antioxidant defense and induces oxidative stress through several mechanisms (for review, see Lopes et al. [6] and Mitra et al. [7]). The generation of free radicals and ROS causes oxidative damage in lipids, proteins, and nucleic acids, culminating in cell injury and tissue dysfunction [8,9,10]. Among these, DNA oxidation is the major concern, as it may induce a variety of damages, including strand breaks and base modifications; a well-written overview of different modes of ROS-induced DNA damage has been published elsewhere [11,12].

Epidemiological data showed that genetic polymorphisms may modulate Pb body burden and, consequently, impact the toxicity of the metal [13,14]. Polymorphisms of the hemochromatosis (HFE) gene are known to cause a mild form of hereditary hemochromatosis, a disease related to a dysregulation of iron (Fe) uptake, increasing the levels of the metal in the body [15]. One hypothesis is that the HFE protein, which is responsible for regulating the absorption of Fe, is also related to the absorption of other essential and toxic metals, including Pb. In this context, variations in the gene encoding the HFE protein could also alter the Pb uptake, modulating the levels of the metal in the body [16]. Although earlier studies showed the impact of HFE polymorphisms on Pb body burden, no studies were carried out to assess the underlying effects of this polymorphism on Pb-induced toxicity.

Therefore, the present study aimed to assess the impact of HFE polymorphisms (rs1799945, C → G; and 1800562, G → A) on blood and plasma lead levels (BLL and PLL, respectively), as well their modulation on DNA oxidative damage induced by Pb, by monitoring of urinary 8-hydroxy-2′-deoxyguanosine (8-OHdG), in Pb-exposed workers from car battery manufacturing factories, in Brazil.

## 2. Material and Methods

### 2.1. Study Population and Blood Collection

This is a cross-sectional study comprising 236 participants (men > 18 years of age) from automotive battery factories, in Paraná State, Brazil, recruited during June and July, 2015. Recruitment of participants was undertaken by a previous visit to the factories to explain the aim of the study. Written consent was given by all participants and this study has received approval for research ethics from Federal University of São Paulo.

For all participants, a questionnaire was administered to collect demographic variables such as age, dietary habits, medical history, consumption of alcoholic beverages, smoking, and occupational exposure. Participants who declared any clinically manifested diseases at the time of recruitment were excluded from the study. Moreover, all participants stated that they used personal protective equipment and followed all the company’s safety regulations, based on the guideline published by the Brazilian Ministry of Health (Health Care for Workers Exposed to Metallic Lead, 2006) [17].

Blood samples were taken on-site in the infirmary of the factories and collected in evacuated tubes (BD Vacutainer; Franklin Lakes, NJ, USA); plasma samples were obtained by centrifugation at 1000× *g* for 10 min. Urine samples were taken in 50 mL conical tubes (Falcon, Corning, NY, USA). Transportation of samples was carried out in dry ice to the laboratory. Samples were stored at −80 °C until further handling.

### 2.2. Blood Lead Levels (BLL) and Plasma Lead Levels (PLL) Determination

BLL and PLL were determined using an inductively coupled plasma mass spectrometer (ICP-MS; ELAN DRC II, Perkin Elmer, Norwalk, CT, USA), by following the method described in Batista et al. [18,19]. Samples were directly diluted (for BLL: 0.20 mL blood sample + 9.8 mL diluent; for PLL: 0.50 mL plasma sample + 9.5 mL diluent (0.010% *m*/*v* Triton X-100 and partially distilled HNO_3_ 14 M 0.5% *v*/*v*)) and injected in the ICP-MS. Standard calibration curve was prepared using matrix matching calibrant by adding 0.20 mL base blood or 0.50 mL base plasma in each calibration solution using the same diluent.

Precision and accuracy of BLL and PLL analyses were determined by analyzing certified reference material QMEQAS07B06 human blood from the National Institute of Public Health of Quebec (INSP, Wolfe, QC, Canada) and SE05-05 caprine serum from the Wadsworth Center—New York Department of Health (NYSDOH, Albany, NY, USA). For BLL, within-run and between-run precisions were 2.1 and 3.5%, respectively, and all obtained results were in agreement with the reference values (39 ± 2.6 µg dL^−1^). For PLL, within-run and between-run precisions were 3.9 and 4.7%, respectively, and all obtained results were in accordance with the reference values (6.8 ± 0.36 µg dL^−1^).

Results are expressed as µg dL^−1^.

### 2.3. Biochemical Parameters Related to the Redox Status

The catalase (CAT) activity was measured in peripheral blood as described by Aebi et al. [20]. This method is based on changes in absorbance at 240 nm due to the CAT-dependent decomposition of H_2_O_2_. The activity of the enzyme was related to hemoglobin (Hb) content (κ g Hg^−1^).

Total thiols (expressed as GSH) concentrations were determined in erythrocytes by addition of 5-5′-dithio-bis(2-nitrobenzoic acid) (DTNB) as described by Ellman et al. [21]. DTNB, a symmetric aryl disulfide, reacts with free thiols to form disulfide plus 2-nitro-5-thiobenzoic acid. The reaction product can be quantified by its absorbance at 412 nm. Results are expressed as μmol mL blood^−1^.

Glutathione peroxidase (GPX) activity was determined in blood spectrophotometrically. This method is based on the oxidation of NADPH, which can be measured as the decrease in absorbance at 340 nm [22]. Results are expressed in nmol NADPH min^−1^ g Hb^−1^.

A commercial kit (Hemoglobina Monotest, Inlab Diagnóstica, São Paulo, Brazil) was used to determine Hb in blood according to the manufacturer’s instructions.

### 2.4. Determination of Urinary Levels of 8-Hydroxy-2′-Deoxyguanosine

Analytical standard of 8-OHdG was purchased from Sigma-Aldrich (>98% purity; St. Louis, MO, USA) and the internal standard ^15^N_5_-8-OHdG was purchased from Cambridge Isotope Laboratories (Andover, MA, USA). Stock standard solutions were prepared in ultra-pure water, stored at low temperatures, and protected from light. Acetonitrile (HPLC grade) was purchased from JT Baker (Phillipsburg, NJ, USA) and acetic acid (HPLC grade) was purchased from Macron Fine Chemicals (Center Valley, PA, USA). High purity de-ionized water (resistivity 18.2 MΩ cm) used throughout the experiment was obtained using a Milli-Q water purification system (Barnstead International, Dubuque, IA, USA).

The urinary concentrations of 8-OHdG were measured using the method described previously by Rocha et al. [23]. In short, 100 μL of urine samples were spiked with 10 ng of labeled internal standard, ^15^N_5_-8-OHdG, diluted up to 0.50 mL with ultra-pure water, mixed and analyzed by high-performance liquid chromatography–tandem mass spectrometry (HPLC-MS/MS).

The HPLC-MS/MS analysis was performed with a Shimadzu Prominence Modular HPLC system (Shimadzu Corporation, Kyoto, Japan) interfaced with an API 3200™ electrospray triple quadrupole mass spectrometer (ESI-MS/MS; Applied Biosystems). The chromatographic analysis was carried out on a Zorbax SB-Aq column (150 mm × 2.1 mm i.d. and 3.5 μm particle size; Santa Clara, CA, USA) serially connected to a Javelin guard column (Betasil C18, 2.1 mm × 20 mm and 5 mm particle size; Thermo Electron Corp.) and acetonitrile (A): water (B), with 0.1% of acetic acid (*v*/*v*) as mobile phase. The target compound was eluted by gradient elution at a flow rate of 300 μL min^−1^ starting at 80% (*v*/*v*) B, held for 2.0 min, decreased to 25% B within 2.0 min (4th min), held for 3 min (7th min), further decreased to 5% B within 3 min (10th min), held for 7 min (17th min), and reverted to 80% at the 17.5th min and held for 2.5 min, with a total run time of 20 min. The mass spectrometry was operated in negative ion mode. The electrospray ionization voltage was set at −4.5 kV. The curtain and collision gas (nitrogen) flow rates were set at 10 and 2 psi, respectively, and the source heater was set at 550 °C. The nebulizer gas (ion source gas 1) and the heater gas (ion source gas 2) were both set at 65 psi. The data acquisition was set at a scan speed of 80 ms and a resolving power of 0.70 FWHM. The injection volume was 10 µL. The following tandem mass spectrometry transition channels were used: 284.2 > 168 for 8-OHdG and 289 > 173 for the internal standard, ^15^N_5_-8-OHdG.

A twenty-point calibration curve at concentrations ranging 0.1–100 ng mL^−1^ was built for 8-OHdG. Isotope labeled internal standard (^15^N_5_-8-OHdG) was employed to compensate for possible matrix effects and analyte losses. The linearity of the calibration plots showed R^2^ values close to unity (>0.98). The limit of detection (LOD) was calculated as 3 times the signal-to-noise ratio and the limit of quantification (LOQ) was set 3.3 times higher than the LOD. The LOD and LOQ were 0.15 and 0.50 ng mL^−1^, respectively.

Urinary creatinine levels were determined by using the kit supplied by ByoSystems SA (Barcelona, Spain) on a clinical biochemistry analyzer (Mindray, model BS-200, Shenzhen, China), according to the manufacturer’s recommendations.

Results are expressed as µg of 8-OHdG per g creatinine (µg g creatinine^−1^).

### 2.5. Genotyping Assays

Genomic DNA (gDNA) was extracted from peripheral blood using the ReliaPrep Blood gDNA Miniprep System (Promega, Wisconsin, WI, USA) according to the manufacturer’s instructions. The quality of the DNA purification was verified by measuring the 260/280 (samples were ≥1.8 and ≤1.9) and 260/230 ratios (all samples > 2.0) (Nanodrop 2000, Invitrogen, Carlsbad, CA, USA). gDNA was quantified by the Qubit dsDNA BR Assay Kit (Invitrogen, California, CA, USA) in a Qubit 3.0 fluorimeter (Invitrogen, Carlsbad, CA, USA). All samples were stored at −20 °C until analyses.

HFE polymorphisms (rs1799945, C → G; and 1800562, G → A) were genotyped by TaqMan assays (Applied Biosystems, Carlsbad, CA, USA) according to the manufacturer’s instructions using an ABI 7500 Fast Real-Time PCR System thermocycler (Applied Biosystems, Foster City, CA, USA).

### 2.6. Data Interpretation

Age, duration of exposure, body mass index (BMI), BLL and PLL, 8-OHdG, CAT, GSH, and GPX were analyzed as continuous variables; alcohol consumption (yes or no), smoking (yes or no); and polymorphisms were classified as categorical variables. Participants who drank alcoholic beverages at least five times per week were considered alcohol users and those who smoke at least five cigarettes per day for the previous five years were classified as active smokers.

Hardy–Weinberg equilibrium (HWE) for polymorphisms was assessed by conventional chi-square test. Moreover, due to their low frequency in individuals with the homozygous variant genotype (<5 individuals), participants were classified as those with the non-variant genotype and carriers of at least one variant allele.

Kolmogorov–Smirnov’s tests were applied to verify the normality distribution of the continuous variables and those that presented skewed distribution were Box-Cox transformed as follows: exposure period (λ = 0.00), BLL (λ = 0.50), PLL (λ = 0.00); CAT (λ = 0.50); GPX (λ = 0.00), and 8-OHdG (λ = 0.50).

General multiple linear models were used to assess the impact of HFE polymorphisms on Pb body burden, adjusted for age, BMI, exposure duration, alcohol, and smoking. Since we have previously published that consumption of milk and dairy products (MDP) could modulate Pb body burden, we also included this variable in the model: participants were classified as low-MDP intake and high-MDP intake (for details, see Gomes et al. [24].

Furthermore, general multiple linear models were also applied to assess the impact of HFE polymorphisms on 8-OHdG, as function of BLL and PLL, adjusted for age, BMI, exposure period, alcohol, and smoking. Moreover, we have earlier reported that exposure to Pb is associated with alterations of biochemical parameters of the redox status, such as GSH and GPX, we also included these variables in the model [24,25].

All analyses were performed in SPSS Statistics 23 software (IBM, Armonk, NY, USA) and the results were considered significant when the *p*-value was ≤ 0.050.

## 3. Results

Sociodemographic characteristics, occupational Pb exposure status, BLL, PLL, redox status conditions, and urinary 8-OHdG concentrations are presented in Table 1.

Participants’ age ranged between 18 and 69 years (mean 38 ± 10 years) and the mean BMI was 27 ± 3.9 kg (m^2^) ^−1^; 16% of participants were active smokers, while 34% declared alcohol consumption regularly. Mean BLL and PLL were 21 ± 12 and 0.60 ± 0.71 μg dL^−1^, respectively. The duration of Pb exposure among participants ranged from 1 month to 27 years. The measured activities of antioxidants enzymes CAT and GPX in blood were 124 ± 59.0 κ g Hb^−1^ min^−1^ and 6.5 ± 3.0 nM NADPH min^−1^ mL blood^−1^; GSH levels ranged from 0.080 to 1.0 µM mL blood^−1^. Mean concentrations of urinary 8-OHdG were 4.0 ± 2.4 µg g creatinine^−1^, with values reaching up to 24 µg g creatinine^−1^.

Table 1 depicts the genotype frequencies of HFE polymorphisms, as well as the variant allele frequencies; allele frequencies for both genotypes are in Hardy–Weinberg Equilibrium (*p* < 0.050).

Table 2 summarizes the impact of HFE polymorphisms on BLL and PLL, adjusted for age, BMI, exposure duration, smoking, alcohol, and MDP intake. A positive correlation between exposure period and BLL was found, i.e., the longer the duration of employment in the factories, the higher the BLL. However, the correlation between the exposure period and PLL did not reach statistical significance (*p* = 0.060). Concerning the relationship between HFE polymorphisms on BLL and PLL, the SNP of HFE rs1799945 influenced PLL; i.e., individuals who carry at least one variant allele (CG + GG) tended to have significantly higher PLL than those with the non-variant genotype, while no associations were found between the SNPs and BLL.

Table 3 presents the effect of HFE polymorphism on urinary 8-OHdG levels, as a function of BLL and PLL (models 1 and 2, respectively). A positive association between 8-OHdG and Pb biomarkers was found; however, statistical significance was reached only with PLL. Moreover, the HFE polymorphism rs1800562 modulated the levels of 8-OHdG, as a function of PLL; i.e., carriers of variant allele (GA) showed higher 8-OHdG concentrations than those with the non-variant genotype (GG). No associations were observed between biochemical parameters related to the redox status (CAT, GSH, and GPX) and 8-OHdG.

## 4. Discussion

The use of Pb in automotive batteries is still a cause for occupational exposure to Pb around the world. Contaminants encountered during the battery-recycling arise from battery components and these include Pb, As, and Cd, which can be released into the soil as solid waste or as wastewater during the separation of components in a water bath. In the battery recycling process, Pb is smelted and refined, which can release toxic vapor and particulate dust [26].

Several studies have reported high levels of BLL in workers from this type of industry; most of these studies were conducted in less developed countries. In our study, mean BLL and PLL were 21 ± 12 and 0.60 ± 0.71 µg/dL, respectively, which may be considered a moderate level of exposure, when compared to results reported in the literature. For instance, studies performed in India, Bangladesh, and Pakistan reported BLL levels of 30 ± 4.1 µg/dL, 65 ± 27 µg/dL, and 69 ± 37 µg/dL, respectively [27,28,29]. Nevertheless, adverse health effects may occur at the level of 10 µg/dL, in adults, according to the National Toxicology Program of the United States [30].

Exposure to metals such as Pb can induce oxidative stress through the generation of ROS. One of the targets of ROS is nucleic acids, and the determination of DNA oxidation, including urinary 8-OHdG levels, has been used as a tool in biomonitoring studies with humans exposed to inorganic and organic toxicants [31,32]. Moreover, epidemiological studies have shown that an increase in guanine hydroxylation is associated with a higher risk for cancer [10,33].

It is of great importance to identify a reliable effect biomarker related to toxic metal exposure that can be used for human biomonitoring studies, as it could provide evidence of early biological events that may predict adverse health outcomes [34]. For this reason, several biomarkers are currently applied to human biomonitoring studies exposed to toxic metals and they are related to DNA damage, antioxidant cell defense, and oxidative stress mechanisms, for example. DNA damage in Pb-exposed workers has been evaluated over the years through different methodologies such as chromosomal aberrations, micronucleus, and comet assays [30,34], and many of these works have demonstrated a positive correlation between occupational exposure and DNA damage; however, analysis of these cytogenetic endpoints is time-consuming, laborious, and needs meticulous techniques. The measurement of urinary 8-OHdG levels is a cost-effective approach, due to its stability in urine, the ease (non-invasive) collection of urine without special treatment or processing prior to storage, the requirement of small volumes, and the possibility of using urine samples from previous studies/biobanks [35].

PLL may better reflect the toxicologically labile fraction of circulatory Pb that is more freely available for exchange with target tissues than the metal in whole blood [36,37]. Studies have also reported an apparent severalfold variation in the relative partitioning of Pb between whole blood and plasma (or serum) for a given whole-blood Pb level. This may reflect inherent differences in the plasma Pb/whole blood Pb partitioning among individuals and/or methodologic challenges associated with the collection and analyses of samples [38]. Our results provided further support concerning these statements, since we observed an association between 8-OHdG and PLL, while this observation was not seen concerning 8-OHdG and BLL.

Few research groups investigated urinary 8-OHdG levels in individuals occupationally exposed to Pb. Pawlas et al. [39] showed that workers exposed to Pb (from a Pb and zinc (Zn) smelter and battery recycling plant) had significantly higher levels of 8-OHdG (41 ± 37 ng g creatinine^−1^; BLL 39 ± 10 µg dL^−1^; PLL 0.15 ± 0.066 µg dL^−1^) than the non-exposed group (25 ± 29 ng g creatinine^−1^; BLL 3.0 ± 2.9 µg dL^−1^; PLL 0.0080 ± 0.011 µg dL^−1^), in Poland. In contrast, Malekirad et al. [40] reported that workers exposed to the metal from a Zn and Pb mine, in Iran, and showed lower levels of blood 8-OHdG than its respective control group (8-OHdG 0.51 ± 0.049 ng mL blood^−1^; BLL 9.6 ± 3.2 µg dL^−1^ vs. 8-OHdG 0.54 ± 0.051 ng mL blood^−1^; BLL 5.1 ± 3.1 µg dL^−1^, respectively); however, it is important to highlight that BLLs found by Malekirad and coworkers [40] in the exposed group are much lower than those found in other biomonitoring studies, as stated above; moreover, the authors observed that several parameters, such as the activity of antioxidant enzymes superoxide dismutase (SOD) and glutathione reductase (GR), and total antioxidant capacity, were significantly higher in the exposed group, suggesting that compensatory effects may explain the lower blood 8-OHdG levels found in these workers.

Some studies did not show associations between Pb exposure and increased oxidative DNA damage. Neitzel et al. [41] assessed Thai workers exposed to toxic metals, such as Pb and Cd, from an e-waste recycling facility and did not observe a significant correlation between BLL and urine lead levels (ULL), and urinary 8-OHdG; it is important to highlight that the levels of Pb exposure were very low (BLL 3.4 ± 2.1 µg dL^−1^; ULL 7.5 ± 7.0 µg g creatinine^−1^) and, therefore, cellular antioxidant defense may be able to scavenge ROS production induced by the metal exposure. In another study, Szymańska-Chabowska et al. [42] assessed the impact of toxic metals, including Pb, in copper (Cu) smelters and found that workers with BLL > (median) 23 µg dL^−1^ showed higher levels of 8-OHdG in serum than the individuals with BLL < 23 µg dL^−1^, as well as the non-exposed groups, providing further support that at low levels of Pb exposure, cellular compensatory effects may counteract the oxidative damage induced by the metal.

The heterogeneity in techniques used to quantify urinary 8-OHdG makes it more difficult to compare data between studies, and chemical quantification methods are recommended as gold standard methods for biomonitoring studies [43]. Different studies indicated that urinary 8-OHdG levels may be affected by age [44], alcohol consumption [31,32], smoking [45], and BMI [46]. On the other hand, Dessie et al. [47] evaluating the association between exposure to heavy metals and oxidative DNA damage showed no correlation between urinary 8-OHdG levels and different demographic characteristics that normally show association such as educational status, alcohol consumption, sex, age, and body weight. Indeed, they observed interactions between different social factors and 8-OHdG, i.e., sex with age, sex with alcohol consumption, and alcohol consumption with education. Our results showed no significant associations with any of these variables, possibly because the chronic exposure to Pb predominates other lifestyle-related factors [48]. In addition, the rates of smoking and alcohol consumption were relatively low in this study, which may be a reason for the lack of significant associations, and further discussion about this issue may be speculative.

Pb-induced toxicity may be modulated by variations in uptake and elimination of the metal due to genetic variations in Pb metabolizing enzymes. In this study, we evaluated the impact of two polymorphisms in the HFE gene (rs179945 and rs1800562) on Pb biomarkers, as well as on 8-OHdG levels. Our findings showed that carriers of the variant G-allele for HFE rs1799945 (CG + GG) tended to have higher PLL than the individuals with the non-variant genotype (CC).

Previous studies showed that HFE polymorphisms are related to alterations in Pb body burden; however, data are still contradictory and appear to be related to the degree of metal exposure, as well as other intrinsic variations, which may include several gene–gene or gene–environment interactions. For example, Szymanska-Chabowska et al. [42] assessed the effect of HFE polymorphisms (rs179945 and rs1800562) on BLL, in Polish workers from a Cu smelter and refinery, and did not find statistically significant differences in BLL stratified by the genotypes (overall BLL 37 ± 9.0 µg dL^−1^); however, when the authors compared those individuals in the third quartile (BLL ≥ 44 µg dL^−1^), workers with the non-variant genotype (GG) showed lower BLL than the individuals with AA genotype (for HFE rs1800562). Similar results were reported in a study with 771 Chinese employees from a Pb smelter company that indicated that carriers of variant G-allele for HFE rs1799945 may be highly vulnerable to Pb toxicity [49]. In contrast, in a recent study of an Indian population of 164 lead-exposed subjects from a Pb alloy manufacturing and battery breaking and recycling facility [50], workers with the non-variant genotype (CC) for HFE rs1799945 were shown to be at risk of higher BLLs than those with the variant allele, i.e., CG (median BLL 57 µg dL^−1^ vs. 50 µg dL^−1^).

We also further assessed the effect of HFE polymorphisms on urinary 8-OHdG levels, as a function of BLL and PLL. Positive associations were found between Pb and 8-OHdG levels. Interestingly, we also observed that HFE rs1800562 polymorphism modulated the oxidative DNA damage induced by the metal; i.e., individuals with the GA genotype tended to have higher urinary 8-OHdG levels than those with the non-variant genotype (GG). Although no direct effects may be related to the HFE gene and oxidative DNA damage, it is important to highlight that several underlying interactions may be related to HFE and Pb body burden, which may impact the toxicity of the metal. One hypothesis for this finding is that high levels of Pb are related to the polymorphic alleles for the HFE gene; in this context, it is notable that since such HFE SNPs directly impact Pb concentrations in the body, one possible explanation for our findings is that high Pb concentrations are directly associated with increased oxidative stress, inducing damage to various macromolecules, such as lipids, proteins, and mostly DNA (for review see Nersesyan et al. [51] and Lopes et al. [6]); it is well known that 8-OHdG is one of the most abundant oxidized metabolites related to DNA-damage induced by oxidative stress [43].

It is well established that the adverse health effects induced by exposure to toxic compounds are also modulated by gene–environment interactions (GEI), which comprise the joint influences of genetic and environmental variables (such as sex, diet, health status, and exposure to chemicals) [52]; this approach of analysis presents some advantages since it is possible to draw scenarios where the genetic effects may increase the risk of toxicity or may be protective, as a function of the exposure. Moreover, it is known that genes may work in concert, and therefore, the effects of one gene can induce compensatory changes in others [53].

## 5. Conclusions

Our findings provide further support concerning the impact of HFE polymorphisms on Pb body burden; to the best of our knowledge, this is the first time that the underlying effects of genetic variations in the HFE gene on oxidative DNA damage induced by Pb exposure have been shown, allowing a better understanding of the molecular mechanisms related to adverse health effects induced by Pb.

## Figures and Tables

**Table 1:** General characteristics of the study population.

Variables	N	Mean ± SD	Median	Quartiles			Range
				25%	50%	75%	
Age (years)	236	38 ± 10	38	30	38	45	18–69
BMI (kg (m^2^)^−1^)	231	27 ± 3.9	26	24	26	29	-
Smoking (yes)	236 (39)	-	-	-	-	-	-
Alcohol (yes)	232 (79)	-	-	-	-	-	-
Milk and dairy products ^a^	232	-	-	-	-	-	-
low intake	103	-	-	-	-	-	-
high intake	129	-	-	-	-	-	-
Exposure duration (months)	236	37 ± 46	19	12	19	48	1.0–324
BLL (µg dL^−1^)	236	21 ± 12	20	12	20	30	1.8–52
PLL (µg dL^−1^)	236	0.60 ± 0.70	0.40	0.17	0.40	0.77	0.10–61
CAT (κ g hemoglobin^−1^ min^−1^	236	124 ± 59.0	112	92	112	139	9.00–486
GSH (µmol mL blood^−1^)	235	0.44 ± 0.14	0.42	0.35	0.43	0.52	0.080–1.0
GPX (nmol NADPH min^−1^ mL blood^−1^)	235	6.5 ± 3.0	5.5	4.5	5.5	8.	0.96–14
8-OHdG (µg g creatinine^−1^)	225	4.0 ± 2.4	3.6	2.7	3.6	4.6	0.69–24
HFE rs1799945 (C → G) ^b^	232	-	-	-	-	-	-
CC	185	-	-	-	-	-	-
CG + GG	47	-	-	-	-	-	-
HFE rs1800562 (G → A) ^c^	232	-	-	-	-	-	-
GG	221	-	-	-	-	-	-
GA	11	-	-	-	-	-	-

^a^ It was considered as “low-MDP intake” individuals who consume ≤3 portions per week, while “high-MDP intake” was classified as those participants that have >3 portions per week. One portion of MDP represents a cup of milk (around 200 mL) or a slice of cheese of about 30–50 g or 150–200 g of yogurt (Gomes et al., 2017). ^b^ Hardy–Weinberg Equilibrium (HWE): χ^2^ = 0.044 (*p* < 0.0010). Minor allele frequency (MAF): 0.11 (allele G); ^c^ Hardy–Weinberg Equilibrium (HWE): χ^2^ = 0.14 (*p* < 0.0010). Minor allele frequency (MAF): 0.020 (allele A).

**Table 2:** Impact of HFE polymorphisms on blood lead levels (BLL) and plasma lead levels (PLL) adjusted for age, body mass index (BMI), exposure period, smoking, alcohol, and milk and dairy product (MDP) intake.

Variables	BLL ^d^		PLL ^c^	
	β ^e^	*p*	β ^e^	*p*
Age	−0.0020	0.75	−0.0010	0.92
BMI	−0.11	0.13	**−0.14**	**0.050**
Smoking ^a^	-	-	-	-
yes	-	-	-	-
no	−0.16	0.39	−0.32	0.080
Alcohol ^a^	-	-	-	-
yes	-	-	-	-
no	0.0020	0.99	0.036	0.79
MDP ^b^				
low intake	0.10	0.44	0.083	0.53
high intake	-	-		
Exposure duration ^c^	**0.11**	**0.045**	0.10	0.060
HFE rs1799945 (C → G)	-	-	-	-
CC	-	-	-	-
CG + GG	0.23	0.17	**0.34**	**0.043**
HFE rs1800562 (G → A)	-	-	-	-
GG	-	-	-	-
GA	−0.40	0.27	−0.31	0.39

^a^ yes was taken as reference; ^b^ high intake was considered as reference; ^c^ Box-Cox transformed (λ = 0.00); ^d^ Box-Cox transformed (λ = 0.50); ^e^ unstandardized beta (β) coefficients for covariates in the model. Math model: BLL or PLL = α + β1×age + β2×BMI + β3 × smoking + β4 × alcohol + β5 × MDP + β6 × exposure period + β7 × HFE (rs1799945) + β8 × HFE (rs1800562). In this case, the statistically significant values are bolded.

**Table 3:** Impact of HFE polymorphisms on urinary 8-OHdG, as a function of blood lead levels (BLL; model 1); and plasma lead levels (PLL; model 2) adjusted for age, body mass index (BMI), exposure period, smoking, alcohol, catalase (CAT), glutathione (GSH), and glutathione peroxidase (GPX).

Variables	Model 1 (BLL)		Model 2 (PLL)	
	8-OHdG ^c^		8-OHdG ^c^	
	β ^d^	*p*	β ^d^	*p*
Age	0.0010	0.91	0.0010	0.92
BMI	−0.0050	0.79	−0.0010	0.94
Smoking ^a^	-	-	-	-
yes	-	-	-	-
no	−0.13	0.50	−0.091	0.62
Alcohol ^a^	-	-	-	-
yes	-	-	-	-
no	−0.073	0.61	−0.080	0.57
Exposure duration ^b^	0.029	0.69	0.018	0.81
BLL ^c^	0.13	0.056	-	-
PLL ^b^	-	-	**0.19**	**0.0060**
CAT ^c^	0.057	0.44	0.058	0.42
GSH	0.28	0.58	0.29	0.57
GPX ^b^	0.037	0.61	0.030	0.67
HFE rs1799945 (C → G)	-	-	-	-
CC	-	-	-	-
CG + GG	0.27	0.11	0.24	0.16
HFE rs1800562 (G → A)	-	-	-	-
GG	-	-	-	-
GA	0.77	0.052	**0.78**	**0.046**

^a^ yes was taken as reference; ^b^ Box-Cox transformed (λ = 0.00); ^c^ Box-Cox transformed (λ = 0.50); ^d^ Unstandardized beta (β) coefficients for covariates in the model. Math model: 8-OHdG = α + β1 × age + β2 × BMI + β3 × smoking + β4 × alcohol + β5 × exposure period + β6 × (BLL) or (PLL) + β7 × CAT + β8 × GSH + β9 × GPX + β10 × HFE (rs1799945) + β11 × HFE (rs1800562). In this case, the statistically significant values are bolded.

## Data Availability

Once requested, the raw data supporting the conclusions of this article will be made available by the authors, without undue reservation.

## Abbreviations

8-OHdG: 8-hydroxy-2′-deoxyguanosine; BLL: blood lead levels; BMI: body mass index; CAT: catalase; Cd: cadmium; DTNB: 5-5′-dithio-bis(2-nitrobenzoic acid; Fe: iron; gDNA: genomic DNA; GEI: gene–environment interactions; GPx: glutathione peroxidase; GSH: total thiols; Hb: hemoglobin; HFE: hemochromatosis; HPLC-MS/MS: high-performance liquid chromatography–tandem mass spectrometry; HWE: Hardy–Weinberg equilibrium; ICP-MS: inductively coupled plasma mass spectrometer; LOD: limit of detection; LOQ: limit of quantification; MDP: milk and dairy products; Pb: lead; PLL: plasma lead levels; ROS: reactive oxygen species; SNP: single nucleotide polymorphisms; ULL: urine lead levels.
